# Pellagra

**Published:** 1916-07

**Authors:** H. C. Buck

**Affiliations:** Friars Point, Miss.


### Pellagra.

BY H. C. BUCK, M. D., FRIARS POINT, MISS.

For many years before pellagra made its appearance in the United States the etiology of the disease occupied the time and close study of many scientific medical men. The cause of this disease and its proper treatment is still occupying the attention of some of the brightest minds of the medical profession, as well as those of the profession who live in sections of our country where this disease is prevalent. The etiology of this disease has not been settled to the satisfaction of any one, so far as my knowledge extends. If one will read all of the many theories advanced as to the cause of this disease, he will become so confused he will probably conclude that no one knows or else all are in a measure correct. There are now a great many able men in the profession who doubt the correctness of the maize theory. Many claim it is an intestinal intoxication or poison. Dr. Goldberg of the United States Public Health Service, in a report recently made and widely circulated in our State (Mississippi), claims, "the inference, therefore, may be safely drawn that pellagra is not an infectious disease, but that it is a disease of essentially dietary origin." There are others who claim the blood is first infected by or through the bite of some blood-sucking insect, notably the sand-fly and buffalo gnat, also the stable fly. The latest theory I have seen is that the disease is caused by a lack of "vitanim" in the system. In justice to Dr. Goldberg, I will say his report is in accord with this theory.

"Vitanim" is a chemical body recently discovered and has been isolated from the hulls of rice and maize, from potatoes and other vegetables, and is present in numerous vegetables and fruits. The writer claims that "Funk of Lister Institute of Preventive Medicine, London," has formulated a definite conception concerning four vexing trophic disturbances, beri-beri, scurvy, rickets and pellagra, and has coined for this group the expressive term "avitaminoses," which is to say pathological states determined by depriving the organism of its needful supply of "vitanim." Another more general name he bestows on this group is "deficiency diseases." In support of Funk's theory, the result of experiments show that first "vitanim" is found in the husk or skin of corn and rice, and where either of these are "milled" and the husk removed beri-beri or pellagra followed its use as food, while on the other hand when the grain is not "milled," or we might say "bolted," but leaving the husk or bran as part of the corn or rice, pellagra or beri-beri did not occur.

In further support of Funk's theory, we can recall the fact that up to a few years before pellagra made its appearance in the United States the people of the South, especially the farmers, sent their corn to country mills and had it ground into meal. When the cook was preparing this for bread it was run through a "sieve" or "sifter" and the coarser part of the husk was removed, but a large part went through the sieve and the "vitanim" remained in the meal and was eaten with the bread. While the people used meal in this condition, there was no pellagra in the country, but a few years after the almost universal use of the bolted meal became prevalent the disease made its appearance and has increased rapidly from year to year. Of course, this theory is new, and in the experimental stage, but to my mind it proves two things: one is, the disease is an intestinal disease, and the other the diet has everything to do with its production.

As to whether the disease is infectious or contagious is still an unsettled question. I believe most authorities contend it is neither. In his report Dr. Goldberg asserts most positively it is not infectious, but is silent as to its contagious properties. My experience with the disease inclines me to believe that it is both.

Two years ago during the winter a negro woman from South Mississippi, where pellagra is very prevalent, moved into my neighborhood, and lived in the house with her parents where there was a large family. The woman was in the third year of pellagra and died in the spring. Up to the time of her arrival all of her father's family were in good health. None of them showed the least indication of having the disease, nor was there any in that immediate locality. But during the summer four members of this family developed pellagra, and the mother now has this disease. To me this has very much the appearance of infection or contagion. We all know it is necessary to know the cause of a disease before intelligent treatment can be adopted.

As to the treatment of pellagra, the general consensus of opinion among physicians is that arsenic in some form is *the*

remedy. My old friend, Dr. J. W. Gray, of Clarksdale, Mississippi, is very successful in treating the disease with tincture of opium. Dr. Goldberg is credited by the newspapers as saying, "by diet it is caused and by diet it can be cured in four weeks." I do not know that Dr. Goldberg made this assertion, nor am I disposed to question the correctness of the theory, but I do know that except in a hospital where one has absolute control of his patient it is impossible to even test it. Especially is this true among the negroes in the South. In a recent issue of a medical journal I read an article wherein the writer said, "Good results are also said to follow the use of living Bulgarian bacilla cultures." Dr. Wm. L. Law says that "in the last five typical cases of pellagra he has treated he prescribed the Bulgarian lactic acid bacilla in tablet form, two tablets being taken half an hour before each meal and at bed time." Dr. Law says: "The improvement in appearance and subjective symptoms of these patients warrant this preliminary report."

Dr. Isadore Dyer, of New Orleans, claims to have had remarkably good results in treating this disease with quinine hydrobromide. In the *Southern Medical Journal* for May, 1916, is an article by Dr. W. F. Cole, of Waco, Texas, wherein he claims to have cured the disease by the administration of calomel, soda and santonin, the old and tried treatment for intestinal worms. So many and varied are the theories and treatments advanced of this disease it is impossible to mention all of them. But if one will read only a few he is forced to the conclusion that the treatment is empirical that no man is able to say just why he uses certain remedies except that some one recommend them or that he has obtained good results by their use.

Personally, I believe that the disease is caused by a toxic poison in the digestive tract, and an intestinal antiseptic is indicated; hence, I give in every case calcium sulphide, and continue the use of this remedy clear through the treatment. To an adult, I give one grain three times a day, and to children dose according to age. In addition, I give Fowler's solution in full doses, alternating each week with iodide potash. When there is diarrhea, I give laudanum with the arsenic; treating other symptoms as they may present themselves. I instruct my patients to confine their diet as much as possible to vegetables and fruits, but to use as little corn in any form as possible. I have had good success with this plan of treatment, but some patients die in spite of any or all treatments. I will add that I begin my treatment in nearly every case by first thoroughly unloading the whole intestinal tract.

[I have known Dr. Buck for over twenty years, and know to an absolute, secret-order certainty that Dr. Buck believes every word that he says he believes. But that does not help me much in judging his paper. He decides that the vitanim theory proves that pellagra is an intestinal disease caused by diet; and later he gives excellent proof to show that he is correct in his opinion that the disease is both infectious and contagious, citing cases. Then he confides that "personally" he believe that the disease is caused by a toxic poison in the digestive tract. Now, I know Buck and have never known him to be entirely wrong.—So take your choice.—Editor.]

